# Multiple mechanisms activate GCN2 eIF2 kinase in response to diverse stress conditions

**DOI:** 10.1093/nar/gkae006

**Published:** 2024-01-28

**Authors:** Jagannath Misra, Kenneth R Carlson, Dan F Spandau, Ronald C Wek

**Affiliations:** Department of Biochemistry and Molecular Biology, Indiana University School of Medicine, 635 Barnhill Drive, MS4067 Indianapolis, Indiana 46202, USA; Department of Biochemistry and Molecular Biology, Indiana University School of Medicine, 635 Barnhill Drive, MS4067 Indianapolis, Indiana 46202, USA; Department of Biochemistry and Molecular Biology, Indiana University School of Medicine, 635 Barnhill Drive, MS4067 Indianapolis, Indiana 46202, USA; Department of Dermatology, Indiana University School of Medicine, 635 Barnhill Drive, MS4067 Indianapolis, Indiana 46202, USA; Richard L. Roudebush Veterans Administration Medical Center, Indiana University School of Medicine, 635 Barnhill Drive, MS4067 Indianapolis, Indiana 46202, USA; Department of Biochemistry and Molecular Biology, Indiana University School of Medicine, 635 Barnhill Drive, MS4067 Indianapolis, Indiana 46202, USA

## Abstract

Diverse environmental insults induce the integrated stress response (ISR), which features eIF2 phosphorylation and translational control that serves to restore protein homeostasis. The eIF2 kinase GCN2 is a first responder in the ISR that is activated by amino acid depletion and other stresses not directly related to nutrients. Two mechanisms are suggested to trigger an ordered process of GCN2 activation during stress: GCN2 monitoring stress via accumulating uncharged tRNAs or by stalled and colliding ribosomes. Our results suggest that while ribosomal collisions are indeed essential for GCN2 activation in response to translational elongation inhibitors, conditions that trigger deacylation of tRNAs activate GCN2 via its direct association with affected tRNAs. Both mechanisms require the GCN2 regulatory domain related to histidyl tRNA synthetases. GCN2 activation by UV irradiation features lowered amino acids and increased uncharged tRNAs and UV-induced ribosome collisions are suggested to be dispensable. We conclude that there are multiple mechanisms that activate GCN2 during diverse stresses.

## Graphical Abstract

**Graphical Abstract:**
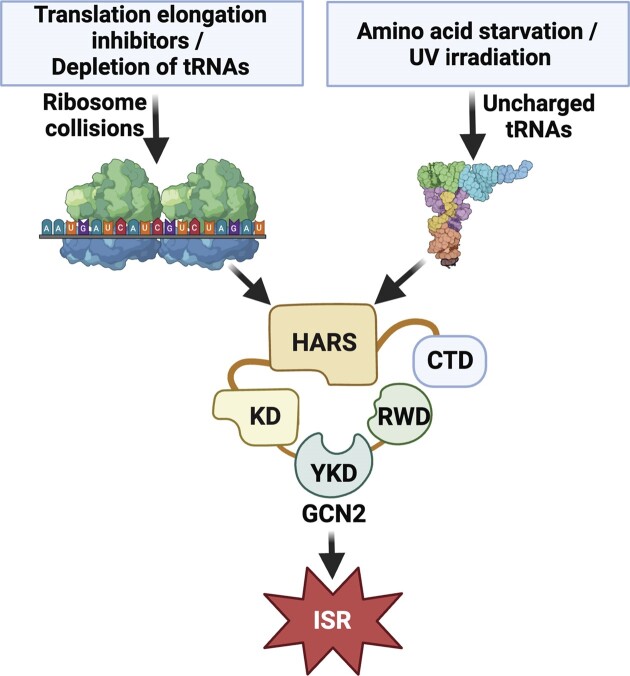

## Introduction

The integrated stress response (ISR) is critical for adaptation to physiological and environmental disturbances of cell homeostasis. The ISR pathway is initiated by the phosphorylation of the α subunit of eukaryotic translation initiation factor 2 (eIF2), which results in the lowering of protein synthesis that enables cells to conserve energy and nutrients and reprogram gene expression to ameliorate stress damage and restore protein homeostasis (1,2). Coincident with the reduction in global protein synthesis, phosphorylation of eIF2α (P-eIF2α) also induces translation of selected stress-adaptive gene transcripts, such as that encoding the ATF4 transcription factor (3–6). In this way, the ISR directs both translational and transcriptional programs of adaptive gene expression, including those designed to restore protein folding and optimize synthesis, uptake, and recycling of amino acids (4–7).

A collection of eIF2α kinases function as first responders in the ISR, each having different regulatory regions that monitor distinct stress conditions (1,8–10). For example, the eIF2α kinase GCN2 (EIF2AK4) is activated by starvation for amino acids, UV irradiation, metabolic perturbations, and oxidative stress conditions (2,8,11). To monitor these diverse cellular stresses, GCN2 has an amino-terminal RWD-protein interaction region, pseudo kinase and protein kinase domains, a region related to the histidyl tRNA synthetase (HARS), and a carboxy-terminal domain (CTD) that can form an interdigitated dimer and can facilitate GCN2 binding to ribosomes. Uncharged tRNAs that accumulate during depletion for different amino acids are suggested to bind to the HARS-related region of GCN2, triggering an ordered mechanism of activation involving trans intra-dimer phosphorylation at the kinase activation loop of GCN2 and eventual P-eIF2α (2,11–15). Engagement of GCN2 with elongating ribosomes is suggested to contribute to its recognition and placement of uncharged tRNAs in the ribosome A site and that process involves enhancing proteins, such as GCN1 (11,16–19).

The mechanisms by which GCN2 is activated by a diverse collection of stresses, some not directly involving accumulation of uncharged tRNAs, is still not yet well resolved. Ishimura *et al.* (20) reported that stalling of elongating ribosomes can be a potent inducer of P-eIF2α by GCN2. More recently, ribosome collisions that can occur with stalling of translating ribosomes were suggested to be a direct or indirect trigger for activation of GCN2 (21). For example, during GCN2 activation by UV-C exposure damaged mRNAs are suggested to create stall points for translating ribosomes that culminate in activation of the protein kinase mitogen-activated protein kinase kinase kinase ZAK (also known as MAP3K20 or MLK7), which then engages with ribosome-associated GCN2/GCN1 complex to induce P-eIF2α by GCN2 (21). Furthermore, treatment of cells with lower concentrations of certain agents that block translation elongation that serve to recapitulate ribosome pausing and collisions were reported to be potent inducers of ZAK-dependent activation of GCN2 (21). Stalled ribosome engagement with GCN2 has also been inferred to contribute to GCN2 activation during nutrient limitation. In *in vitro* and cell culture models it was reported that ribosome P-stalk proteins, uL-10 and P1, which are adjacent to the A site can directly engage with GCN2 and at least in purified models be more potent activating ligands of GCN2 than uncharged tRNAs (22,23).

Here we investigated the role of uncharged tRNAs and ribosome collisions in GCN2 activation in response to different stress arrangements, including treatment with translation elongation inhibitors, agents that block aminoacylation of tRNAs or selected depletion of a target tRNA, and UV-B or UV-C irradiation. Our results suggest that ZAK and ribosomal collisions are indeed essential for GCN2 activation in response to treatment with translational elongation inhibitors. Consistent with the direct tRNA engagement model, we suggest that GCN2 activation by accumulating cognate uncharged tRNAs occurs by direct binding with the GCN2 HARS-related domain and ZAK and ribosome collisions are not required. Furthermore, in human keratinocytes exposed to UV-B or UV-C, depletion of amino acids and elevated uncharged tRNAs are also linked with GCN2 activation and ZAK and ribosome collisions are largely dispensable for GCN2 induction. Our results indicate that there are multiple mechanisms that activate GCN2 during diverse stresses, some processes involve direct engagement with uncharged tRNAs, while others require ribosome collisions.

## Materials and methods

### Cell culture and treatment regimens

Human embryonic kidney cells with SV40 T antigen (HEK293T) were purchased from ATCC (#CRL-3216). Cells were grown in Dulbecco's Modified Eagle Medium media (DMEM, Corning, #10-013-CV) supplemented with 10% (v/v) fetal bovine serum (Corning, #35-010-CV), 100 units/ml penicillin and 100 μg/ml streptomycin (Cytiva, #SV30010). An immortalized human keratinocyte cell line, NTERT, which has been shown to have normal differentiation properties (24–29), was maintained and passaged in low calcium EpiLife Complete media (Gibco, #MEPI500CA) supplemented with human keratinocyte growth supplement (HKGS; Themo Fisher Scientificc, #S0015) and 1000 U Penicillin-Streptomycin (PS) (Gibco Laboratories, #15140122) as previously described (28). Mouse embryonic fibroblast (MEF) cells were cultured in DMEM supplemented with 10% FBS as previously described (30). HeLa-CCL-2 cells were purchased from ATCC and cultured in EMEM following the supplier guidelines. Treatment doses and times for anisomycin (Sigma-Aldrich, #A9789), blasticidin (Gibco, #R210-01), halofuginone (HF) (Cayman Chemical Co., #13370), borrelidin (Tocris, #4706), histidinol (Sigma-Aldrich, #H6647), velcrin (DNMDP) (MedChemExpress, #HY-W028690), puromycin (Calbiochem, #CAS 58-58-2), cycloheximide (Sigma-Aldrich, #C7698), MEM Amino Acids Solution (50X) (Gibco, #11130051), non-essential amino acids (NEAA) 100X Solution (Cytiva HyClone, #SH30238.01), N-Acetyl-L-cysteine (NAC) (Sigma-Aldrich, #A9165), CellROX^®^ Green Reagent (Invitrogen, #C10444), and yeast tRNA^Phe^ (Sigma, #R4018) were added as described in the results and figure legends. For collective supplementation of cystine, glycine, and serine, the final concentrations were 1 μM, 10 μg/ml, and 30 μg/ml, respectively. After a 30-min culture period in the supplemented medium, the NTERT cells were subjected to 400 J/m^2^ UV-B and cultured for an additional 3 h.

UV-B irradiation was carried out using Philips FS20T12 UV-B broadband light sources as described previously (31). An IL1700 radiometer and an SED240 UV-B detector (International Light, Newburyport, MA) were used to measure UV-B intensity prior to each experiment, from a distance of 8 cm from the light source to the culture dish. UV-C irradiation of NTERT keratinocytes was conducted using an Ushio G20T10 UV-C light sources. An ILT77 germicidal radiometer and UV-C detector (International Light, Newburyport, MA) was used to measure the UV-C intensity prior to each experiment. To control for the influence of phenol red on UV-induced oxidative stress, HEK293T cells were transferred from DMEM media to PBS before UV irradiation and then returned to DMEM media afterward; whereas NTERTs were UV-irradiated in EpiLife Complete media which does not contain phenol red.

### Plasmid constructions

The pCas-Guide-AAVS1(Cat #GE100023) and pAAVS1-EF1a-Puro-DNR (Cat #GE100046) plasmids were obtained from Origene. A segment of DNA encoding a FRT site and eGFP CDS were sub cloned into pAAVS1-EF1a-Puro-DNR using an In-Fusion HD cloning kit (Takara, #638920) to produce pAAVS1-EF1a-FRT-eGFP-Puro-DNR. Flp recombinase expression plasmid pCAG-Flpe (plasmid #13787) was purchased from Addgene (32). Plasmid pCDNA5-FRT was purchased from Invitrogen (Cat #V601020) and GCN2 cDNA plasmid pENTR223-1 GCN2 (NM_001013703) was purchased from Transomic Technologies (Huntsville, AL). The cDNA encoding the human *GCN2* coding sequence was sub-cloned from pENTR223-1 GCN2 into pCDNA5-FRT and an amino-terminal 3x FLAG tag was added using an In-Fusion HD cloning kit in a three-way cloning reaction. The resulting plasmid pCDNA5-FRT-GCN2 is referred to in the text as FLAG-WT-GCN2. This plasmid was used as a template to construct the mutant version pCDNA5-FRT-GCN2-m2 by site directed mutagenesis using an In-Fusion HD cloning kit (33). This mutant plasmid is referred to in the text as FLAG-GCN2-m2 with TCC/AGG → CTG/CTG encoding residue substitutions F1143L/R1144L.

### Generation of knockout cell lines by CRISPR

Deletion of *ZAK* in HEK293T cells was achieved by CRISPR using a pCas-Guide vector (Origene, #GE100002) and linear donor DNA containing LoxP-EF1A-tGFP-P2A-Puro-LoxP (Origene, #KN402432D). Briefly, a human *ZAK* guide (sense) with the sequence 5′-ATGGATATCACAGGACAAGG-3′ was inserted into pCas-Guide vector. HEK293T cells were then co-transfected with pCas-guide vector containing the human *ZAK* guide sequence and a linear donor LoxP-EF1a-GFP-P2A-Puro-LoxP using lipofectamine 3000 transfection reagent (Invitrogen, #L3000008) following the manufacturer's protocol with a 3:1 ratio of lipofectamine 3000 to plasmid DNA. 48 hours post transfection, the cells were divided into a 1:10 ratio and given 3 more days to grow. The cultured cells were then again split 1:10, and this process was repeated 4 times in total. For the final passage, cells were seeded at a density of 1 cell per well in a 96-well plate. The wells contained complete media supplemented with puromycin at a final concentration of 1 μg/ml. Puromycin was added to select puromycin-resistant clones, as the linear donor carries the puromycin-resistant gene. Cells were maintained in this condition until colonies of cells were readily visible. The colonies were then expanded and screened for loss of *ZAK* mRNA expression by qRT-PCR and ZAK protein by immunoblot analyses.

Deletion of *ZAK* in NTERT cells was created by using the Alt-R CRISPR-Cas9 system (IDT, #Hs.Cas9.MAP3K20.1.AA). Briefly, ribonucleoprotein (RNP) complex was formed by mixing human *ZAK*-specific CRISPR RNA (5′-TCGAGCCAAATGGATATCAC-3′, in Exon 2 of ZAK), a transactivating crRNA (tracrRNA), and Cas9 endonuclease following the manufacturer's guidelines. The gene specific CRISPR RNA hybridizes with the tracrRNA forming the guide RNA that leads the Cas9 endonuclease to the desired location of the genome for cleavage. The RNP complex was then transfected into low passage wild type NTERT cells by electroporation. Cells were then plated onto a 10 cm dish and allowed to form colonies which were expanded and screened for *ZAK* expression by qRT-PCR and immunoblot analyses.

Stable expression of amino terminal tagged WT GCN2 and GCN2-m2 proteins were carried out by FLP-FRT recombination (34) in combination with GCN2-derived plasmids described in the plasmid constructions section of the Materials and methods. We began with generation of HEK293T-FRT cells by introducing a cassette containing FRT-eGFP-Puro^r^ by CRISPR/Cas9 into the *AAVS1* locus of HEK293T cells following a modification of previously reported strategy (35). Briefly, cells were co-transfected with pCas-Guide-AAVS1 and pAAVS1-EF1a-FRT-eGFP-Puro-DNR plasmids then selected with 1 μg/ml puromycin in DMEM. The pAAVS1-EF1a-FRT-eGFP-Puro-DNR plasmid contains two 500-bp sequence segments homologous to the *AAVS1* locus to allow for recombination following cleavage by Cas9. Successful incorporation of the cassette imparts the cells with eGFP expression and puro resistance and introduces an Flp recombinase target site (33).

Deletion of *GCN2* was achieved in the HEK293T-FRT cells using the CRISPR/Cas9 Human Gene Knockout Kit (Origene, # KN412459). Briefly, 0.2 × 10^6^ cells were seeded into 6-well tissue culture plates and 1 μg of a plasmid pCas-Guide-GCN2 plasmid encoding Cas9 and a sgRNA (5′-GGACTTCCAAGACCTGCGGC-3′) were transfected using FuGENE 6 reagent into the cells. Transfections were carried out for 24 h with 3:1 ratio of FuGENE to plasmid DNA. The transfected cells were passaged 5 times over the course of 10 days, and cells were then seeded into 10 cm plates at a density of 200 cells/plate for colony formation. Cells were maintained in DMEM for 7–10 days with media change every other day until cell colonies were visibly identified. Colonies were then expanded and screened for GCN2 expression by immunoblot analysis. The GCN2 KO cells were confirmed by sequencing of the genomic DNA segment of the GCN2 gene, along with the loss of expressed GCN2 protein (33).

Cell lines stably expressing FLAG-WT-GCN2 were generated from HEK293T-FRT-GCN2 KO cells by Flp recombinase mediated integration following the following adaptation of a published protocol (34). Successful recombination inserts *GCN2* and Hygro^r^ genes from the pCDNA-FRT-GCN2 plasmid into the FRT site integrated at the *AAVS1* locus while separating the eGFP coding sequence from its translational start site. The resulting cells are hygromycin resistant and eGFP negative. HEK293T-FRT-GCN2 KO cells were seeded into 6-well plates at 0.2 × 10^6^ cells/well then co-transfected with 1.8 μg pCAG-Flpe and 0.2 μg pCDNA5-FRT-GCN2 plasmids. Forty-eight hours post-transfection, cells were split into 10 cm plates and stable transfectants were selected for using 200 μg/ml hygromycin-B for 10 days. Hygromycin resistant colonies that lacked eGFP expression were then expanded and screened for FLAG-GCN2 expression by immunoblot analyses. Insertion of FLAG-GCN2 at the *AAVS1* locus was verified by PCR from genomic DNA (33). A FLAG-GCN2-m2 with substitutions F1143L/R1144L in the HARS-related domain was created in parallel in the HEK293T-FRT-GCN2 KO using the same protocol. The siRNA-mediated knock down of GCN2 in HeLa cells was achieved using the following GCN2 siRNAs: siGCN2 #1: 5′-GCAAUUCUGUGGUGCAUAA-3′ (Dharmacon, #J-005314-06) and siGCN2 #2: 5′-GGAAAUUGCUAGUUUGUCA-3′ (Dharmacon, #J-005314-08).

### Immunoblot analyses

Lysates were prepared from cells using a solution containing 1% SDS supplemented with 1X Halt protease and phosphatase inhibitor cocktail (Thermo Scientific, #1861281). Lysates were boiled for 5 minutes, sonicated using a Branson Sonifier, and clarified by centrifugation at 12 000 × g. Protein concentrations were measured using a DC protein assay kit (Biorad, #5000112) using BSA as a standard. Equal amounts of protein lysates were separated by electrophoresis in SDS gels and transferred onto a 0.2 μm nitrocellulose membrane using a Trans-Blot Turbo RTA nitrocellulose transfer kit (Biorad, #1704270). Target proteins and their primary antibodies with vendor information and experimental dilutions that were used in the immunoblot analyses include ZAK 1:1000 (Bethyl, #A301-993A), P-GCN2 (T899) 1:1000 (Abcam, #75836), total GCN2 1:1000 (Cell Signaling, #3302), P-eIF2α (S51) 1:1000 (Abcam, #32157), total eIF2α 1:1000 (Cell Signaling, #5342), P-p38 MAPK 1:2000 (Cell Signaling, #4511S), total p38 MAPK 1:2000 (Cell Signaling, #8690S), CReP 1:1000 (Proteintech, # 14634-1-AP), Actin 1:5000 (Sigma, #A5441). Following incubation with primary antibodies at room temperature, membranes with bound proteins were washed three times for 10 minutes with TBS-T buffer solution and then incubated with 1:5000 GAR-HRP (Biorad, #170-6515) or GAM-HRP (Biorad, #170-6516) secondary antibodies for 45 min and washed again three times with TBS-T buffer solution for 10 minutes. Immunoblot signals were visualized with ECL solution (1:1 mixture of 100 mM Tris (pH 8.5), 2.5 mM luminol, 0.4 mM P- coumaric acid: 100 mM Tris (pH 8.5), 0.06% H_2_O_2_ vol/vol) or SuperSignal West Femto Maximum Sensitivity Substrate (Thermo Scientific, #34094); images were captured using a Chemidoc MP imaging system (Biorad).

### Ribosome collision assays

After the indicated treatment regimens, cells were washed once with PBS and then collected in 500 μl of lysis buffer solution (20 mM Tris·HCl [pH 7.5], 100 mM NaCl, 10 mM MgCl_2_, and 0.4% NP-40) containing 50 μg/ml cycloheximide and 25 Unit/ml Turbo DNase 1 by vigorously scraping. Subsequently, the cells were passed through a 23 G needle 8–10 times, and the resulting lysates were incubated on ice for 10 min. The cell lysates were clarified by centrifugation for 10 min at 15000 xg at 4°C, and the supernatant was transferred to new microfuge tubes. Cell lysates were then digested with 100 units of RNase I (Ambion, #AM2295, 100 Unit/μl) at 4°C for 1 h with mild agitation. The digestions were halted wih the addition of 200 units of SUPERase·IN (Ambion, #AM2696, 20 unit/μl) were added. The digested samples were then layered onto 10–50% sucrose gradients supplemented with SUPERase·IN and subjected to ultracentrifugation in a Beckman SW41Ti rotor at 40 000 rpm for 2 h at 4°C. After centrifugation, a piston gradient fractionator (BioComp) and a 254 nm UV monitor with Data Quest Software were used to generate profiles.

### Genome-wide tRNA-charging assays

Measurements of charged tRNAs genome-wide were carried out using a high-throughput tRNA charging assay, which relies on the simultaneous sequencing of both charged and uncharged tRNA libraries. Following treatment of cells as indicated, total RNA was extracted from cells using TRIzol (Life Technologies, 15596018). 2 μg of total RNA for each sample was then treated with 12.5 mM NaIO_4_ (oxidized) in sodium acetate buffer (pH 4.5) in the dark at room temperature for 20 min followed by quenching with 0.3 M glucose for 10 min. All mature tRNAs end with 3′-CCA. NaIO4 will only oxidize the 3′-A residues of uncharged tRNAs since the 3′ end of charged tRNAs are protected by linked amino acids; total RNA was then passed through MicroSpin G-50 column (GE Healthcare, #27533001) for desalination. Next, tRNA was discharged (deacylation through β-elimination) by keeping the total RNA in a solution of 50 mM Tris–HCl (pH 9.0) at 37°C for 45 min, followed by quenching with a solution containing 50 mM sodium acetate buffer (pH 4.5) and 100 mM NaCl. Total RNA was then recovered by an overnight EtOH precipitation at −20°C. The tRNA samples were then treated with T4 polynucleotide kinase (New England BioLabs, #M0201) to remove the 3′-PO_4_ group from the uncharged tRNA. T4 polynucleotide kinase treatment is necessary because following oxidation, β-elimination in the basic condition selectively removed the oxidized 3′-A residue, leaving a 3′-PO_4_ at the terminal 3′-C residue for uncharged tRNAs, whereas β- elimination removes the amino acid attached to the 3′-A residue of charged tRNAs, resulting in 3′-A-OH for these charged tRNAs. The presence of 3′-PO_4_ at the terminal 3′-C of uncharged tRNAs will block them from being ligated to the 5′-adenylated adaptor for downstream processing, therefore removal of this PO_4_ group is necessary. Next, 5′-adenylated adaptors carrying unique barcode (5′-PO_4_-NNNNNXXXXXAGATCGGAAGAGCACACGTCTGAA-ddC-3′; N-random nucleotides, XXXXX- is sample specific pentamer barcode, ddc-dideoxy cytidine) were ligated to the tRNAs using T4 RNA ligase2 truncated KQ (New England BioLabs, #M0351L). tRNAs were then ligated to a 5′-adaptor (5′-rGrUrUrCrArGrArGrUrUrCrUrArCrArGrUrCrCrGrArCrGrArUrC-3′) followed by reverse transcription using SuperScript IV RT kit (Invitrogen, #18090050) and RT primer (5′-AGACGTGTGCTCTTCCGATCT-3′). Next the tRNA library was PCR amplified for 10 cycles by using Illumina multiplex PCR primer (5′-AATGATACGGCGACCACCGAGATCTACACGTTCAGAGTTCTACAGTCCGA-3′) and Illumina barcode PCR Primer (5′-CAAGCAGAAGACGGCATACGAGATGCCTAAGTGACTGGAGTTCAGACGTGTGCTCTTCCGATCT-3′). PCR products were separated by an 8% non-denaturing polyacrylamide TBE gel (Invitrogen, #EC6215BOX) and ∼210 nt bands were excised, purified and subjected to HiSeq paired-end Illumina sequencing.

Fastq files for the pooled samples were demultiplexed according to the five-nucleotide sample barcode contained within the ligated adapter using a custom python script. We have uploaded the demultiplexing python script and the rest of the analysis scripts to Figshare (https://doi.org/10.6084/m9.figshare.24553186.v1). Pairs without an identifiable barcode could not be matched to a sample and were thus discarded. Illumina Sequencing adapters and ligated sample barcode adapters were trimmed using cutadapt in paired read mode and reads with less than twenty nucleotides remaining after trimming, corresponding to empty adapters, were removed. A custom reference file for human tRNA was constructed from the gtRNAdb GRCh38 high confidence tRNA gene set by removing duplicate sequences and appending ‘CCA’ to the 3′-end of each unique tRNA gene sequence. Trimmed reads were then aligned to the custom tRNA reference file using bowtie2 short read aligner with options ‘–dovetail -D 20 -R 3 -N 1 -L 20 -i S,1,0.50’.

The charging status of the aligned tRNA was determined from the 3′-end of the aligned portion of each read pair, where reads ending in ‘CCA’ were determined to be charged and reads ending in ‘CC’ were determined to be uncharged. Counts of charged and uncharged tRNA for each tRNA gene in each sample were then imported to R for further analysis. Differences in read counts between samples were accounted for by multiplying the read counts in each sample by a normalization coefficient equal to the average number of reads across all samples divided by the read count in that sample. The fraction charged for each tRNA isodecoder within each sample was then determined summing the normalized counts of charged and uncharged reads for each gene corresponding to that isodecoder, and the mean was then averaged for each of replicate of each treatment. The mean fraction charged for each isodecoder was then visualized as a heatmap and barplot ± S.D. of the mean using ggplot2. Statistical significance in difference of the mean fraction charged was determined using a Welch's *t*-test with Benjamini–Hochberg FDR correction to account for repeated measures. The genome-wide tRNA-charging assay data generated in this study have been deposited in the NCBI’s GEO database under the accession code GSE239867. Total and uncharged levels of specific tRNAs were also measured by qPCR similar to that previously described (36,37). The following primers were used for qPCR (presented 5′ to 3′): human tRNA^Leu^_(TAA)_fw-GAGTGGTTAAGGCGTTGGAC,rev-GAGAATTCCATGGTCGAACCCACG; yeast tRNA^Phe^fw-GCGGAYTTAGCTCAGTTGGGAGAG, rev-GAGAATTCCATGGTGCGAAYTCTGTGG; human tRNA^Pro^fw-GGCTCGTTGGTCTAGGGGTA, rev-GAGAATTCCATGGGGGCTCGTCCG; human tRNA^Asn^fw-GTCTCTGTGGCGCAATCGGT, rev-GAGAATTCCATGGCGTCCCTGG.

### Measurements of GCN2 binding to tRNAs

The GCN2 KO HEK293T cells or GCN2 KO HEK293T cells expressing either FLAG-WT-GCN2 or FLAG-GCN2-m2 were seeded at a density of 3 × 10^6^ cells per 10 cm plate. After treatment, the plates were placed on ice, washed once with ice-cold PBS, and then 500 μl of lysis buffer (25 mM Tris pH 7.6, 150 mM NaCl, 1 mM EDTA, 1% NP-40, 5% glycerol) supplemented with 1× Halt protease inhibitor was added to each plate and incubated on ice for 5 min. Cells were then collected with a cell scraper and transferred to the 1.5 ml Eppendorf tubes. Samples were clarified by centrifugation in a microfuge at 13000 xg for 10 min at 4°C, and the supernatant was transferred to a new Eppendorf tube. Concentrations of each lysate sample was measured by NanoDrop using lysis buffer as the blank. In parallel with the lysate preparation, 50 μl Anti-FLAG bead slurry was transferred to 1.5 ml Eppendorf tubes (one tube for each lysate preparation). Beads were washed 3× with 500 μl lysis buffer solution, and the beads were collected each time with a magnetic stand for subsequent incubation with clarified cell lysates. For each sample lysate containing equal amount of protein was added to the magnetic beads and incubated on a nutator for 20 min at room temperature. After incubation, the beads were collected with a magnetic stand and washed twice with 500 μl of PBS and once with nuclease-free water. RNA was then extracted with TRIzol RNA extraction reagent and dissolved in nuclease-free water.

Next tRNA associated with the FLAG-WT-GCN2, FLAG-GCN2-m2, or control GCN2 KO cells was detected by qRT-PCR as previously described (36). Briefly, isolated RNAs were oxidized followed by spiking with yeast tRNA^Phe^ for internal normalization, followed by deacylation at high pH. Following deacylation, RNA samples were treated with polynucleotide kinase (New England BioLabs, #M0201), followed by ligation to adapters using RNA ligase 2 truncated KQ (New England BioLabs, #M0351L). The resulting tRNAs were then annealed to an RT primer and converted to cDNA using a SuperScript IV RT kit (Invitrogen, #18090050). The following primers were used for qPCR (presented 5′ to 3′): yeast tRNA^Phe^ fw-GCGGAYTTAGCTCAGTTGGGAGAG, rev-GAGAATTCCATGGTGCGAAYTCTGTGG; human tRNA^Pro^ fw-GGCTCGTTGGTCTAGGGGTA, rev-GAGAATTCCATGGGGGCTCGTCCG; human tRNA^Asn^ fw-GTCTCTGTGGCGCAATCGGT, rev-GAGAATTCCATGGCGTCCCTGG. The data was normalized to FLAG-WT-GCN2 control and presented as fold change.

### Polysome profiling assays

NTERT cells were treated with either 400 J/m^2^ UV-B or left untreated. Following recovery for 3 h, cycloheximide was added to each culture dish at a final concentration of 50 μg/ml for 10 min before harvesting. Cells were rinsed with ice-cold PBS solution containing 50 μg/ml cycloheximide and then lysed with 500 μl of cold lysis solution containing 20 mM Tris·HCl (pH 7.5), 100 mM NaCl, 10 mM MgCl_2_, 0.4% NP-40, and 50 μg/ml cycloheximide, followed by centrifugation at 15 000 × g for 10 min at 4°C. Cell lysates were then applied to the top of 10–50% sucrose gradients and subjected to ultracentrifugation in a Beckman SW41Ti rotor at 40 000 rpm for 2 h at 4°C. A piston gradient fractionator (BioComp) and a 254 nm UV monitor with Data Quest Software were used to generate whole cell lysate polysome profiles as described previously (38).

### Amino acid measurements

Levels of free amino acids were measured in cultured cells by liquid chromatography–tandem mass spectrometry (LC–MS). Cell pellets were resuspended in 0.1% formic acid in methanol, vortexed briefly, and subjected to three cycles of freezing in liquid nitrogen followed by thawing at 42°C for 10 min. Clarified lysates were analyzed by LC–MS/MS with stable isotope dilution. Lysates were spiked with a mixture containing 37 amino acid isotopic internal standards and deproteinated using a solution of 90% acetonitrile, 10 mM ammonium formate, and 0.15% formic acid. Clarified lysates were analyzed using a biphasic LC–MS/MS approach first with a HILIC separation (39) followed by a mixed-mode chromatographic separation (40). Quantification of amino acid levels was determined by fitting response ratios to an eight-point calibration curve generated using verified reference material for each of the 20 amino acids quantified.

### ROS and GSH measurements

The NTERT cells were cultured in EpiLife Complete media supplemented with HKGS and penicillin-streptomycin as detailed above in the Cell culture and treatment regimens of the Materials and methods. For supplementation of additional amino acids both MEM Amino Acids Solution 50× (Gibco, #11130051) and Non-Essential Amino Acids (NEAA) 100× (Cytiva HyClone, #SH30238.01) solution were supplemented to the EpiLife Complete media, each at final levels of 3×. Alternatively, 5 mM NAC (Sigma-Aldrich, #A9165) was supplemented into the EpiLife Complete media. After a 30-min culture period in the supplemented medium, the NTERT cells were subject to 400 J/m^2^ UV-B and cultured for an additional 3 h. ROS levels were measured by CellROX Green reagent according to the manufacturer's protocol. GSH levels were measured by GSH/GSSG Ratio Detection Assay Kit (Abcam, #ab138881) according to the manufacturer's protocol.

### Statistical analyses

One-way or two-way analysis of variance (ANOVA) or unpaired two-tailed Student's *t*-tests were used to calculate statistical significance, as indicated. *P* values <0.05 were considered statistically significant and significance in figures was indicated as follows: *P ≤ 0.05, **P ≤ 0.01, ***P ≤ 0.001, ^****^P ≤ 0.0001. Results were expressed as the standard deviation (SD) of the mean and are representative of at least three biological replicates.

## Results

**Figure 1.**
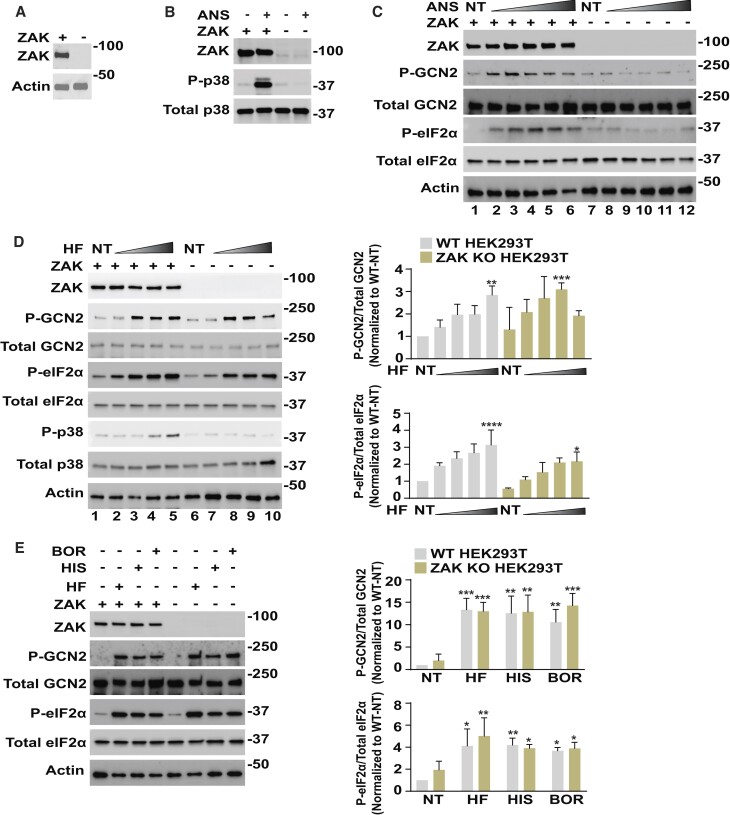
Activation of GCN2 by agents that invoke translation inhibition. (**A**) *ZAK* was deleted by genome editing and CRISPR and WT and ZAK KO cells, designated + and -, respectively, were cultured and ZAK and actin proteins were measured in the prepared lysates by immunoblot analyses with antibodies that specifically recognize each protein. Molecular weight (MW) markers are indicated in kDal. (**B**) WT HEK293T cells (+) and those deleted for ZAK (−) were treated with 1 μM anisomycin (ANS) for 1 h (+) or not treated (−), followed by immunoblot measurements of the phosphorylated and total p38 proteins, along with ZAK. (**C**) WT (+, lanes 1–6) and ZAK (−, lanes 7–12) cells were treated with up to 200 nM ANS for 3 h. Levels of the indicated phosphorylated and total proteins were measured by immunoblot analyses. ANS treatment for lanes: 1 and 7 (0 nM, not treated -NT), 2 and 8 (5 nM), 3 and 9 (25 nM), 4 and 10 (50 nM), 5 and 11 (100 nM), and 6 and 12 (200 nM). (**D**) WT (+, lanes 1–5) and ZAK KO (−, lanes 6–10) HEK293T cells were treated with increasing amounts of HF for 3 h, followed by immunoblot measurements of the indicated phosphorylated and total proteins (left panel). HF treatment for lanes: 1 and 6 (0 nM, not treated -NT), 2 and 7 (5 nM), 3 and 8 (25 nM), 4 and 9 (50 nM), and 5 and 10 (100 nM). Relative levels of P-GCN2 and P-eIF2α normalized for the respectively total protein (P-total) are shown in the bar graph (right panel). The P/total for GCN2 and eIF2α proteins was normalized to the respective NT-WT group and presented as fold change. (**E**) WT (+) and ZAK KO (−) HEK293T cells were treated with 25 nM HF, 4 mM histidinol (HIS), or 2 μM borrelidin (BOR) for 3 h, followed by immunoblot measurements of the indicated phosphorylated and total proteins (left panel). Relative levels of P-GCN2 and P-eIF2α normalized for total levels of the corresponding proteins are shown in the bar graph (right panel). The P/total ratio for GCN2 and eIF2α proteins was normalized to the respective NT-WT group and presented as fold change. For panels D and E, statistical significance, denoted by *, was determined using a two-way analysis of variance (ANOVA); *P ≤ 0.05, **P ≤ 0.01, ***P ≤ 0.001, ^****^P ≤ 0.0001. Error bars indicate mean with SD (*n* = 3).

Lower amounts of agents that inhibit translation elongation, such as anisomycin, can create ribosome collisions that induce ZAK, which in turn activates GCN2 in the ISR (21). Expression of ZAK was omitted in HEK293T cells by genome editing and CRISPR and wild-type (WT) and ZAK knockout (KO) cells were treated with either anisomycin or vehicle (Figure 1A and B). Anisomycin treatment increased p38 phosphorylation (P-p38), a downstream substrate of ZAK protein kinase, in WT cells but not in the ZAK KO cells, validating the functional loss of ZAK. Next, we treated these cells with increasing doses of anisomycin. Lower concentrations of anisomycin, with an optimum about 25 nM, activated GCN2 in WT cells as measured by self-phosphorylation (P-GCN2) and by P-eIF2α (Figure 1C). By contrast, cells deleted for ZAK showed minimal activation of GCN2 and P-eIF2α in response to anisomycin treatment. Furthermore, activation of GCN2 at lower concentrations of another translation inhibitor blasticidin also triggered robust P-GCN2 and P-eIF2α in HEK293T and mouse embryo fibroblast (MEF) cells (Supplementary Figure S1A and B). These results are consistent with a prior report that ribosome collisions triggered by treatment with certain translation elongation inhibitors activate GCN2 and the ISR by mechanisms requiring ZAK (21).

### Activation of GCN2 by accumulating uncharged tRNAs does not require ZAK

Halofuginone (HF) is a febrifugine derivative that potently inhibits prolyl tRNA synthetase activity associated with the bifunctional EPRS protein, leading to rapid accumulation of uncharged tRNA^Pro^ and activation of GCN2 (36,41). We treated the WT and ZAK KO HEK293T cells with increasing concentrations of HF and determined that there were enhanced levels of P-GCN2 and P-eIF2α that occurred independent of ZAK function (Figure 1D). By comparison, at higher levels of HF (100 nM) there was enhanced P-p38 that was sharply diminished in the ZAK KO cells, which suggests that these concentrations of HF can trigger ribosome collisions. The requirement of ZAK for HF induction of P-p38 would be consistent with our prior report that HF can trigger pausing of elongating ribosomes at proline codons as measured by ribosome profiling (36). ZAK was also dispensable for induction of P-GCN2 and P-eIF2α in the HEK293T cells treated with the agents histidinol and borrelidin, which specifically lowers charging of tRNA^His^ and tRNA^Thr^, respectively (Figure 1E). These results suggest that in these cultured cells that ZAK is not required for activation of GCN2 by accumulating levels of multiple different uncharged tRNAs.

### Ribosome collisions facilitate GCN2 activation by elongation inhibitors but are dispensable in response to accumulating uncharged tRNA

**Figure 2.**
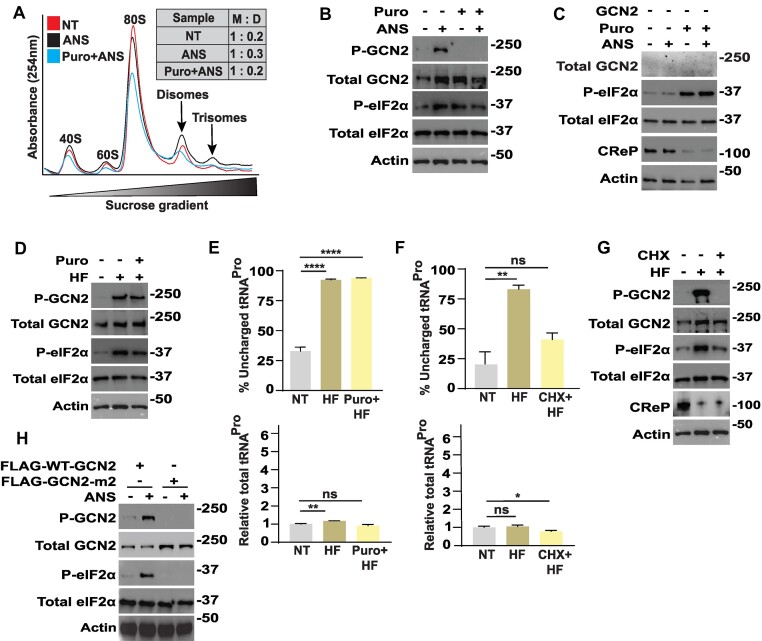
Role of ribosome collisions in the activation of GCN2. (**A**) WT HEK293T cells were either not treated (NT) or treated with either 25 nM anisomycin (ANS) alone or in combination with 1μM puromycin (Puro) for 3 h. Lysates were prepared, treated with RNase I, and subjected to sucrose gradient centrifugation and fractionation. Ribosomes were visualized by measuring the absorbance at 254nm. The profile indicates the free 40S and 60S ribosomal subunits, 80S monosomes and colliding translating ribosomes, which are represented by disomes and trisomes. M- monosomes, D-disomes. (**B**) WT HEK293T cells were not treated (−) or treated with 25 nM ANS in presence or absence of 1 μM Puro for 3 hours, as indicated (+). The designated phosphorylated and total proteins were measured by immunoblot analyses. (**C**) GCN2 KO HEK293T cells were not treated (−) or treated with 25 nM ANS in presence or absence of Puro for 3 h, as indicated (+), followed by immunoblot analyses with the indicated antibodies. (**D**) WT HEK293T cells were not treated (−) or treated with 25 nM HF in presence or absence of Puro for 3 h, as indicated (+), followed by immunoblot measurements of the specified phosphorylated and total proteins. (**E**) WT HEK293T cells were treated with 25 nM HF in the presence or absence of 1 μM Puro for 3 h or not treated (NT), as indicated. The percentage of uncharged tRNA^Pro^ (top panel) and levels of total tRNA^Pro^ relative to NT were measured by RT-qPCR and represented in the bar graph (bottom panel). Statistical significance was determined using a one-way analysis of variance (ANOVA); Significance is indicated by **P ≤ 0.01 and ^****^P ≤ 0.0001 and ns indicates not significant difference. Error bars indicate mean with SD (*N* = 3). (**F**) WT HEK293T cells were treated with 25 nM HF in presence or absence of 50 μg/ml cycloheximide (CHX) for 3 h or not treated (NT). The percentage of uncharged tRNA^Pro^ (top panel) and relative levels of total tRNA^Pro^ normalized to the NT group (bottom panel) were measured by RT-qPCR and represented in the bar graph. Statistical significance was determined using a one-way analysis of variance (ANOVA); Significant is indicated by **P ≤ 0.01 and *P ≤ 0.05, and ns indicates no significant differences. Error bars indicate mean with SD (*n* = 3). (**G**) WT HEK293T cells were not treated (−) or treated with 25 nM HF in presence or absence of 50 μg/ml cyclohexmide (CHX) for 3 h, as indicated (+). Lysates were prepared and the specified phosphorylated and total proteins were measured by immunoblot analyses. (**H**) HEK293T cells stably expressing only FLAG-WT-GCN2 or FLAG-GCN2-m2 were treated with 25 nM ANS (+) or no treatment (−) for 3 h, followed by measurements of phosphorylated or total GCN2 and eIF2α by immunoblot analyses.

Cell treatment with lower concentrations of anisomycin cause ribosome stalling that leads to collisions between translating ribosomes and ZAK-mediated GCN2 activation (21). The ribosome collisions can be measured by first treating lysates with non-specific RNase I, followed by visualization of ribosomes by sucrose gradient centrifugation. Consistent with a prior study showing ribosome collisions (21), cells treated with anisomycin displayed appreciable disomes and more modest amounts of trisomes that are resistant to the RNase I (Figure 2A).

Puromycin is an aminonucleoside antibiotic and tyrosyl-tRNA analog that is incorporated into nascent polypeptides and results in premature ribosome release of both the nascent polypeptides and bound mRNA. We reasoned that puromycin pre-treatment would reduce ribosome collisions and ensuing GCN2 activation. We pretreated the WT HEK293T cells with puromycin, followed by the GCN2-activating dose of anisomycin, and as predicted the puromycin pretreatment lowered the accumulation of disomes and trisomes (Figure 2A). It is noted that there is partial reduction of disomes albeit comparable to no stress treatment, which is consistent with a previous report suggesting that puromycin does not as potently release ribosomes that are elongating slowly due to an occupied A site or translocation inhibition (42). Nonetheless, the puromycin combined with anisomycin potently lowered activation of GCN2 as measured by P-GCN2 (Figure 2B). This finding was recapitulated in MEF cells, where blasticidin activation of GCN2, as measured by P-GCN2, was largely thwarted with the puromycin pretreatment (Supplementary Figure S2). These results support the idea that ribosome collisions triggered by certain elongation inhibitors are critical for activation of GCN2.

It is noteworthy that although P-GCN2 was sharply lowered by the combined treatments of puromycin and anisomycin, there was continued elevated amounts of P-eIF2α that was also viewed with puromycin treatment alone (Figure 2B). Puromycin induction of P-eIF2α occurred even in cells deleted for GCN2 (Figure 2C). Previous studies reported that agents that block translation elongation can induce P-eIF2α (30). A reason for this induction involves the expression of the CReP, which targets Type I protein phosphatase for dephosphorylation of P-eIF2α (43). Expression of the short-lived CReP is rapidly depleted during robust inhibition of protein synthesis, such as treatment with puromycin of the GCN2 KO cells (Figure 2C). The lowered CReP expression would contribute to enhanced P-eIF2α even if there was no appreciable increase in eIF2α kinase activities. These results explain the discordant levels of P-GCN2 and P-eIF2α in the combined anisomycin and puromycin assays and emphasize that P-GCN2 is the more reliable measure of GCN2 activity.

### Ribosome collisions are not required for activation of GCN2 by accumulating uncharged tRNAs

To address whether HF activation of GCN2 requires ribosome collision, we treated WT HEK293T cells with HF in absence or presence of puromycin pretreatment. In contrast to anisomycin, activation of GCN2 by HF occurred even when combined with puromycin (Figure 2D). Furthermore, as expected HF triggered accumulation of uncharged tRNA^Pro^ that occurred independent of puromycin pretreatment (Figure 2E). Levels of total tRNA^Pro^ were similar among the treatment groups.

We also carried out analogous experiments with cycloheximide, which blocks the translocation step of translation elongation. When HF treatment of HEK293T cells was combined with cycloheximide pretreatment there was lowered accumulation of tRNA^Pro^ (Figure 2F). Here, we reasoned that the block in protein synthesis by cycloheximide would sharply lower the expenditure of charged tRNA^Pro^ and thus diminish the cell requirement for prolyl tRNA synthetase-catalyzed replenishment. In the example of puromycin, it is suggested that there is appreciable synthesis of truncated nascent polypeptides that when combined with HF still allows for HF depletion of charged tRNA^Pro^. As a consequence, the cycloheximide pretreatment eliminates HF activation of GCN2 as measured by self-phosphorylation (Figure 2G). As noted earlier for puromycin combined treatments, there was still appreciable levels of P-eIF2α with the coupled HF and cycloheximide treatments, which is suggested to be a result of diminished CReP expression that occurs with the cycloheximide-directed block in translation (Figure 2G). These results support the idea that accumulating uncharged tRNAs can activate GCN2 even with lowered ribosome collisions.

To evaluate the role of the HARS-related domain of GCN2 in the activation of GCN2 by anisomycin, we created a knockout of GCN2 (GCN2 KO) in HEK293T cells and introduced either FLAG-WT-GCN2 or a mutant version FLAG-GCN2-m2, which harbors residue substitutions in the HARS-related domain that blocks yeast GCN2 binding to tRNA (16,44). The WT-GCN2 and GCN2-m2 proteins showed similar levels of expression (Figure 2H). There was robust P-GCN2 and P-eIF2α in response to anisomycin treatment of cells expressing WT-GCN2, and both were diminished in those containing GCN2-m2 (Figure 2H). These results suggests that GCN2 activation by ribosome collisions require a functional HARS-related domain.

### Uncharged tRNA^Pro^ interacts with GCN2 during activation by HF

**Figure 3.**
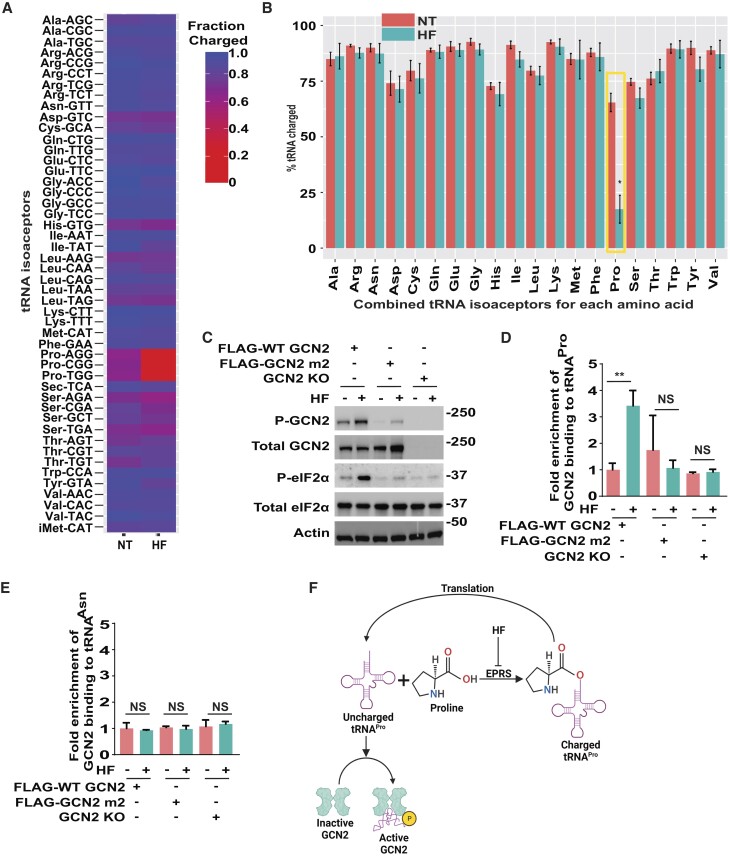
GCN2 binds tRNA^Pro^ in response to HF treatment. (A and B) To measure charging of tRNAs genome-wide, MEF cells were either treated with a 100 nM HF for 3 hours or not treated (NT). Only the tRNA isoacceptors measured in the MEF cells by the genome-wide assay are shown. The percentage of charged tRNAs is shown as a heatmap (**A**) and combined tRNA isoacceptors for each cognate amino acid as a bar graph (**B**). The error bars represent the SD of the mean (*N* = 3). *Indicates *P* ≤ 0.05 determined using a two-sided Welch's *t*-test followed by correction for multiple hypothesis testing using Benjamini–Hochberg FDR method. (**C**) HEK293T cells stably expressing only FLAG-WT-GCN2 or FLAG-GCN2-m2, or GCN2 KO HEK293T cells, were treated with 25 nM HF (+) or no treatment (–) for 3 hours, followed by measurements of phosphorylated or total GCN2 and eIF2α by immunoblot analyses. (D and E) HEK293T cells expressing FLAG-WT-GCN2 or FLAG-GCN2-m2, or GCN2 KO HEK293T cells, were treated with 25 nM HF (+) for 3 hours or not treated (–). Cell lysates were prepared and FLAG-WT-GCN2 and FLAG-GCN2-m2 were pulled down using anti-FLAG antibody linked with magnetic beads. The associated RNA was purified and bound tRNA^Pro^ (**D**) and tRNA^Asn^ (**E**) were measured by qRT-PCR. The bar graph shows fold enrichment for tRNA bound to GCN2 (WT/m^2^) in response to HF (+) or no treatment (–) and is normalized to FLAG-WT-GCN2-no treatment group. Statistical significance was determined using a two-way analysis of variance (ANOVA); **P ≤ 0.01 and ns indicates no significant difference. Error bars indicate mean with SD (*n* = 3). ns-not significant. (**F**) Model for activation of GCN2 by accumulating tRNA^Pro^ in response to HF treatment.

We carried out genome-wide tRNA-charging assays to address the full complement of changes in tRNA charging by HF treatment. In this experiment we used MEF cells that we reported earlier decreased charging of tRNA^Pro^ and activation of GCN2 and translational control in response to HF treatment (36). In the genome-wide measure of tRNA charging, each of the three proline tRNA isoacceptors (tRNA^Pro^_(AGG)_, tRNA^Pro^_(CGG)_ and tRNA^Pro^_(TGG)_) trended lower in response to HF treatment (Figure 3A, Supplementary Figure S3). When proline codons were combined together in the tRNA charging analysis, the decrease in aminoacylation of tRNA^Pro^ was significant upon HF treatment, whereas no other tRNA charging was significantly affected (Figure 3B). These results are consistent with reports that HF is a potent and selective inhibitor of charging of tRNA^Pro^ (36,41).

To evaluate the role of the HARS-related domain of GCN2 in the activation of GCN2 and tRNA binding, we treated FLAG-WT-GCN2 or FLAG-GCN2-m2 with HF. The WT GCN2 and GCN2-m2 proteins showed similar levels of expression, whereas the eIF2α kinase was absent in the GCN2 KO cells (Figure 3C). As expected, there was robust P-GCN2 and P-eIF2α in response to HF treatment of cells expressing WT GCN2, and both were diminished in those containing GCN2-m2 and in GCN2 KO cells (Figure 3C).

Next, we pulled-down the FLAG-WT-GCN2 proteins in the HEK293T cells treated with HF or no stress agent with anti-FLAG antibodies and measured associated tRNAs by qRT-PCR. There was a significant enrichment for tRNA^Pro^ binding with WT GCN2 in response to HF treatment, whereas there was minimal association between tRNA and GCN2-m2 protein that was comparable to the GCN2 KO cells (Figure 3D). There was no association between WT GCN2 and tRNA^Asn^, supporting the specificity of the tRNA binding during HF treatment (Figure 3E). These results support the model that specific uncharged tRNAs bind to the HARS-related domain of GCN2 facilitates activation during nutrient conditions mimicked by HF (Figure 3F).

### Depletion of specific tRNA triggers ribosome collisions and activation of GCN2

**Figure 4.**
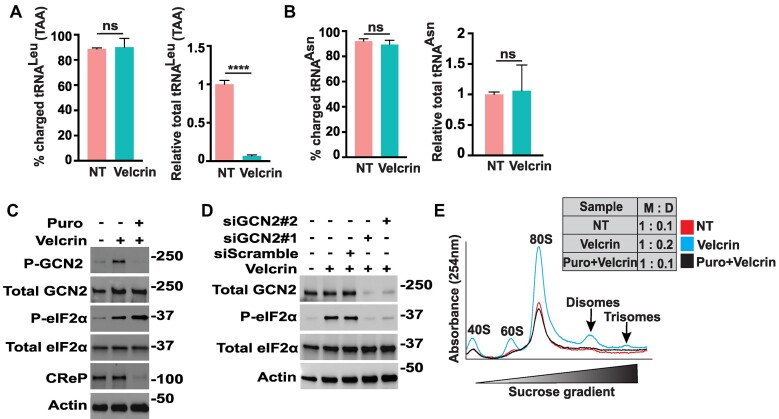
Velcrin activates GCN2 by ribosome stalling. (A, B) HeLa cells were treated with 1 μM velcrin or not treated (NT), as indicated. (**A**) The percentage of charged and total tRNA^Leu^_(TAA)_ was measured by RT-qPCR and the related levels of total tRNA^Leu^_(TAA)_ and the percentage of charged tRNA^Leu^_(TAA)_ are represented in a bar graph. Statistical significance was determined using an unpaired two-tailed *t*-test; ^****^P ≤ 0.0001, error bars indicate mean with SD (*n* = 3), ns indicates not significantly different. (**B**) As a control the levels of total tRNA^Asn^ and percentage of charged tRNA^Asn^ were measured by RT-qPCR and represented in a bar graph. Error bars indicate mean with SD (*n* = 3), ns denotes no significant difference. (**C**) HeLa cells were not treated or treated with 1 μM velcrin alone or in combination with 1μM puromycin (Puro) for 3 h, as indicated by the + and − symbols. Lysates were prepared and the levels of the indicated phosphorylated and total proteins were measured by immunoblot analyses. (**D**) HeLa cells were transiently transfected with one of two different siRNAs targeting depletion of GCN2, or scrambled siRNA, as indicated (+ or −). Transfected cells were treated with treated with 1 μM velcrin for 3 h as indicated (+) and the levels of phosphorylated or total GCN2 and eIF2α were measured by immunoblot analyses. (**E**) HeLa cells were not treated or treated with either 1 μM velcrin alone or in combination with 1 μM Puro for 3 h, as indicated by the index. Polysome profiles from RNase I-digested lysates of these treated cells were generated, with the indicated free 40S and 60S ribosomal subunits, 80S monosomes and colliding translating ribosomes, which are represented by disomes and trisomes. Relative levels of monsomes (M) and disomes (D) are indicated in the inset.

An anticancer agent velcrin was reported to associate with phosphodiesterase 3A (PDE3A) and SLFN12, culminating in this complex selectively cleaving and depleting tRNA^Leu^_(TAA)_ (45). In agreement with this prior study, velcrin treatment of HeLa cells, which were reported to express elevated levels of PDE3A and SLFN12, significantly depleted tRNA^Leu^_(TAA)_ levels without altering its charged fraction (Figure 4A). We did not see any significant changes in either total or charged forms of tRNA^Asn^ which we used as a negative control (Figure 4B). Next, we treated HeLa cells with velcrin and determined that there was robust activation of GCN2 as measured by P-GCN2 and P-eIF2α (Figure 4C and D). Targeted depletion of GCN2 by siRNAs lowered P-eIF2α further indicating that velcrin was a potent activator of GCN2 (Figure 4D).

To evaluate the role of ribosome collisions in the velcrin induction of GCN2, we treated the WT HeLa cells with velcrin alone or in combination with puromycin pretreatment. Whereas velcrin induced P-GCN2, there was minimal activation of the eIF2α kinase when the cells were treated with both velcrin and puromycin (Figure 4C). Consistent with our earlier description of combined puromycin treatments, P-eIF2α was enhanced concomitant with lowered CReP expression, further supporting the idea that P-GCN2 is the optimal measure of GCN2 activation (Figure 4C). Finally, we measured ribosomes collisions during velcrin treatment by treating lysates with RNase I, followed by visualization of ribosomes by sucrose gradient centrifugation (Figure 4E). Velcrin elicited appreciable disosomes and more modest amounts of trisomes, which were largely eliminated with the combined puromycin treatment. These results indicate that depletion of specific tRNAs can trigger ribosome collisions that contribute to activation of GCN2. It is emphasized that the velcrin treatment does not increase uncharged tRNAs (Figure 4A), suggesting that the processes linked with ribosome stalling and collisions are sufficient to induce GCN2.

### UV activation of GCN2 in keratinocytes does not require ZAK

GCN2 is the predominant eIF2α kinase activated by UV irradiation in mammalian cells (46,47). Previous studies in human epithelial MCF10A cells suggested that translation of mRNAs damaged by UV-C irradiation triggered ribosome collisions, facilitating ZAK-directed induction of GCN2 (21). To address the processes of GCN2 activation by UV irradiation, WT HEK293T cells and those depleted for ZAK were exposed to up to 1000 J/m^2^ UV-B. Following 3 h of recovery, which is optimal for GCN2 activity, there was enhanced P-GCN2 and P-eIF2α in the WT cells that was similar to that observed for those depleted for ZAK (Supplementary Figure S4A). Furthermore, at higher doses of UV-B exposure there was enhanced P-p38 that was not detectable in the ZAK-depleted cells. These results suggest that ZAK is not essential for activation of GCN2 in HEK293T cells in response to UV-B exposure.

**Figure 5.**
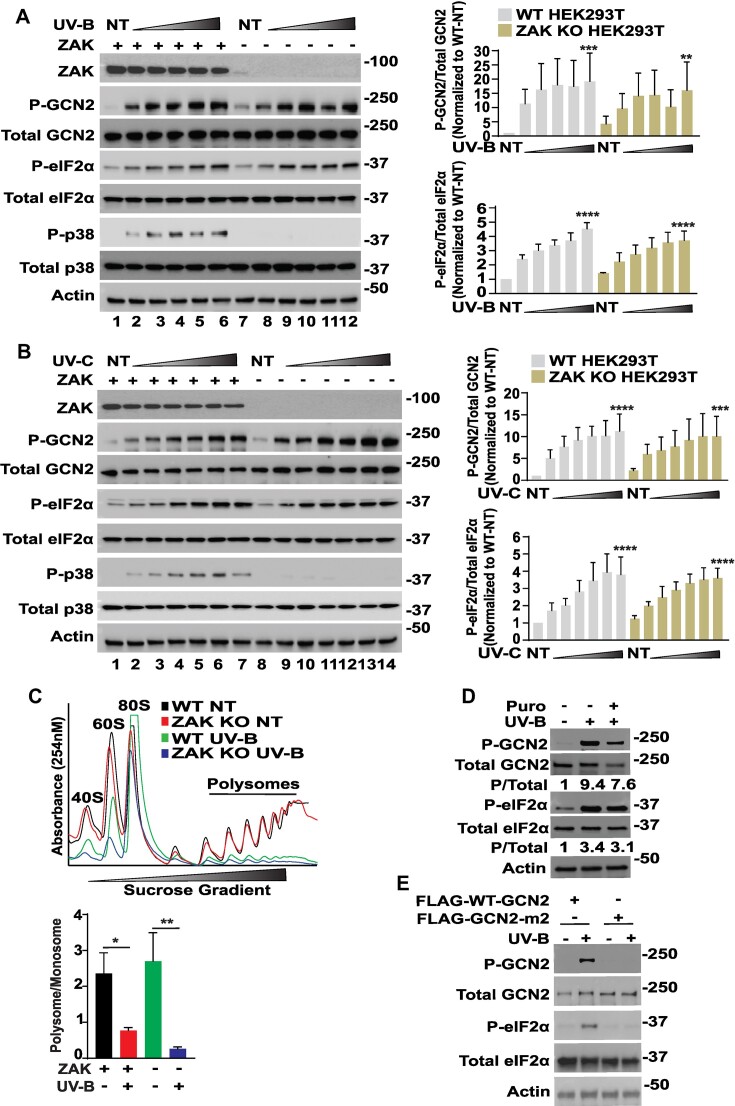
ZAK is not required for UV activation of GCN2 in human keratinocytes. (**A**) Human keratinocyte NTERT cells expressing ZAK (+, lanes 1–6) or deleted for ZAK (-, lanes 7–12) were treated with up to 1000 J/m^2^ of UV-B (**A**) or not treated (NT), followed by 3 h of culture. Protein lysates were prepared and the indicated phosphorylated and total proteins were measured by immunoblot analyses. UV-B treatment for lanes: 1 and 7 (0 J/m^2^), 2 and 8 (200 J/m^2^), 3 and 9 (400 J/m^2^), 4 and 10 (600 J/m^2^). 5 and 11 (800 J/m^2^) and 6 and 12 (1000 J/m^2^). Relative levels of P-GCN2 and P-eIF2α are shown in the bar graph (right panels). P/total ratio for GCN2 and eIF2α proteins was normalized to the respective not treated (NT) WT group and presented as fold change. Statistical significance was determined using a two-way analysis of variance (ANOVA); Significance is indicated as **P ≤ 0.01, ***P ≤ 0.001, ^****^P ≤ 0.0001. Error bars indicate mean with SD (*n* = 3). (**B**) NTERT cells expressing ZAK (+, lanes 1–7) or deleted for ZAK (−, lanes 8–14) were treated with up to 1200 J/M^2^ of UV-C or left untreated (NT), followed by 3 h of culture. The indicated phosphorylated and total proteins were measured by determined by immunoblot. UV-C treatment for lanes: 1 and 8 (0 J/m^2^), 2 and 9 (20 J/m^2^), 3 and 10 (75 J/m^2^), 4 and 11 (150 J/m^2^). 5 and 12 (300 J/m^2^), 6 and 13 (600 J/m^2^), and 7 and 14 (1200 J/m^2^). Relative levels of P-GCN2 and P-eIF2α are shown in the bar graphs (right panel). P/total ratio for GCN2 and eIF2α proteins was normalized to the respective not treated (NT) WT group and presented as fold change. Statistical significance was determined using a two-way analysis of variance (ANOVA); Statistical significance is indicated as ***P ≤ 0.001, ^****^P ≤ 0.0001. Error bars indicate Mean with SD (*N* = 3). (**C**) WT (+) and ZAK KO (−) NTERT cells were treated with a 400 J/m^2^ UV-B or left untreated, followed by recovery for 3 h. Cells were collected, lysed, and analyzed by centrifugation in sucrose gradients. Gradients were fractionated and ribosomes monitored at *A*_254_ nm. Free 40S and 60S ribosomal subunits, 80S monosomes, and polysomes are indicated in the figures. Polysome to monosome ratios were determined for each group and presented in a bar graph (bottom panel). Statistical significance was determined using a two-way analysis of variance (ANOVA); Statistical significance is indicated as *P ≤ 0.05, **P ≤ 0.01. Error bars indicate mean with SD (*N* = 3). (**D**) WT NTERT cells were either left untreated or treated with 400 J/m^2^ UV-B in presence or absence of 1 μM puromycin (Puro) for 3 h, as indicated, followed by immunoblot measurements of the indicated phosphorylated and total proteins. Phosphorylated GCN2 or eIF2α normalized to total protein are presented for each treatment arrangement, with a value of 1 indicated for the sample not treated with either UV-B or Puro. (**E**) HEK293T cells stably expressing only FLAG-WT-GCN2 or FLAG-GCN2-m2 were treated with 400 J/m^2^ UV-B or not treated (−) for 3 h, followed by measurements of phosphorylated or total GCN2 and eIF2α by immunoblot analyses.

Given that human skin is the physiological tissue exposed to UV irradiation from sun light, we next measured UV-induced activation of GCN2 activation in a human keratinocyte cell line designated NTERT and those deleted for ZAK by CRISPR. WT and ZAK deleted NTERTs were treated with UV-B and C in a dose dependent manner followed by 3 h recovery. There were enhanced amounts P-GCN2 and P-eIF2α in response to increasing doses of either UV-B or UV-C and this occurred largely independent of ZAK (Figure 5A and B). By comparison, induced levels of P-p38 were only visible in the WT NTERT cells following either treatment regimen, with minimal P-p38 in ZAK KO cells (Figure 5A and B). We observed similar results with a shorter recovery period (30 min) following UV irradiation (Supplementary Figure S4B and C).

Next, we addressed whether ZAK-deleted keratinocytes undergo translational control similar to WT cells upon treatment with UV irradiation. Exposure to UV-B sharply lowered the polysome to monosome ratio in both wild type and ZAK-deleted cells, consistent with diminished translation initiation (Figure 5C). These results indicate that ZAK is dispensable for activation of GCN2 and attendant translational control in keratinocyte cells exposed to UV. To address whether UV-B activation of GCN2 requires ribosome collision, we treated WT NTERT cells with UV-B in absence or presence of puromycin. In contrast to anisomycin, activation of GCN2 by UV-B occurred even when combined with puromycin (Figure 5D). Similar to anisomycin, UV-B treatment failed to activate mutant version GCN2-m2 in the HEK293T cell model (Figure 5E). These results suggest that both ZAK and ribosome collisions are not essential for GCN2 activation by UV-B irradiation.

### UV-B irradiation depletes cellular free amino acid levels and decreases charged tRNA^Ser^ levels

**Figure 6.**
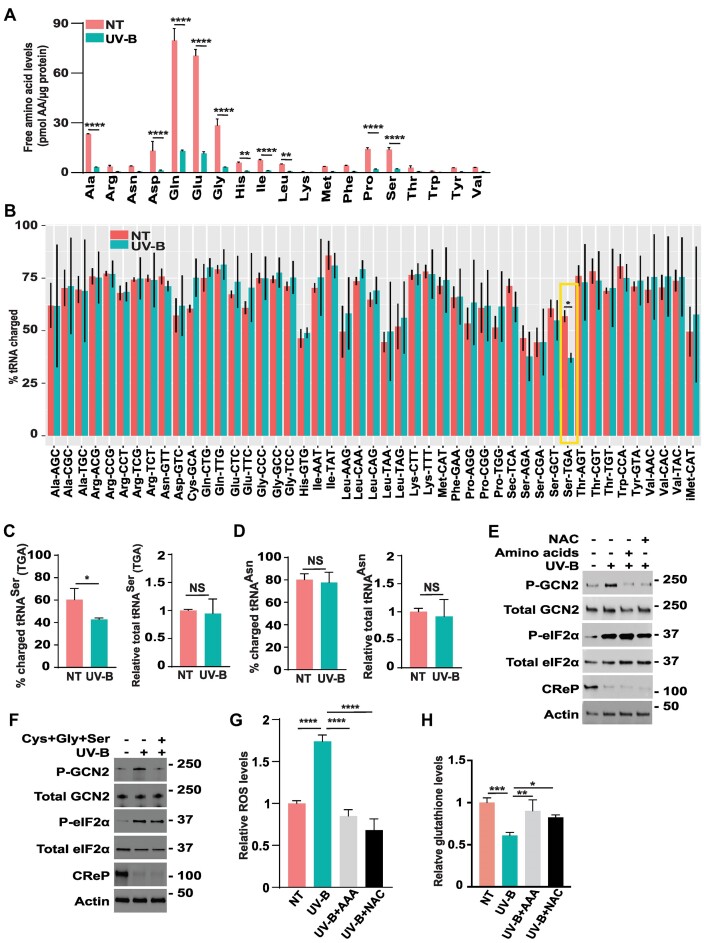
UV irradiation decreases free amino acid levels and lowers charging of tRNA^Ser^. (**A**) WT NTERT cells were treated with a 400 J/m^2^ UV-B or not treated (NT), followed by recovery for 3 h. Cells were lysed, and free amino acids were measured and illustrated in the bar graph. Statistical significance, denoted by *, was determined using a two-way analysis of variance (ANOVA); **P ≤ 0.01, ^****^P ≤ 0.0001. Error bars indicate Mean with SD (*N* = 3) (**B**) Genome-wide charging of tRNAs in WT NTERT cells was determined by genome-wide tRNA-charging assay. Cells were treated with 400 J/m^2^ UV-B or left untreated (NT), followed by recovery for 3 h. Only the tRNA isoacceptors in the NTERT cells that were measured in the genome-wide assay are shown. The percentage of charged tRNA isoacceptors is shown as a bar graph. The error bars represent SD of the mean (*n* = 3). *Indicates *P*-value < 0.05 of two-sided Welch's t-test followed by correction for multiple hypothesis testing using Benjamini-Hochberg FDR method. (**C**, **D**) WT NTERT cells were treated with 400 J/m^2^ UV-B or not treated (NT), followed by recovery for 3 h. The percentage of uncharged of tRNA^Ser^_(TGA)_ (C) and tRNA^Asn^ (D) were measured by a RT-qPCR, along with the relative levels of these tRNAs. Statistical significance was determined using an unpaired two-tailed t-test; *P ≤ 0.05, Error bars indicate mean with SD (n = 3), NS-not significantly different. (**E**) WT NTERT cells were not treated (−) or treated with 400 J/m^2^ UV-B (+) and cultured in medium supplemented with additional amino acids or *N*-acetyl-l-cysteine (NAC), as indicated) for 3 h. Protein lysates were prepared, and the designated phosphorylated and total proteins were measured by immunoblot analyses. (**F**) WT NTERT cells were not treated (−) or treated with 400 J/m^2^ UV-B (+) and cultured in medium supplemented with additional cystine, glycine and serine for 3 h. Protein lysates were prepared, and the designated phosphorylated and total proteins were measured by immunoblot analyses. (**G**) WT NTERT cells were cultured in medium supplemented with additional amino acids or NAC and then treated with 400 J/m^2^ UV-B. Following 3 h of culture, cellular ROS levels were measured by CellROX® Green Reagent. Levels of ROS levels are presented in the bar graph relative to untreated cells. Statistical significance was determined using a one-way analysis of variance (ANOVA); ^****^P ≤ 0.0001. Error bars indicate mean with SD (*N* = 3). (**H**) WT NTERT cells were cultured in medium supplemented with additional amino acids or NAC and then treated with 400 J/m^2^ UV-B or no treatment (NT). Following 3 h of culture, cellular glutathione (GSH) levels were measured and presented in the bar graph relative to NT. Statistical significance was determined using a one-way analysis of variance (ANOVA); *P ≤ 0.05, **P ≤ 0.01, ***P ≤ 0.001. Error bars indicate mean with SD (*n* = 3).

To investigate the effect of UV-B irradiation on the levels of cellular amino acid levels, we exposed the WT keratinocytes to UV-B and determined that the levels of each amino acid trended lower, with the amounts of lowered Ala, Asp, Gln, Glu, Gly, His, Ile, Leu, Pro and Ser achieving significance (Figure 6A). Given that accumulating uncharged tRNA can be a primary signal activating GCN2, we next treated the WT NTERT cells with UV-B and measured aminoacylation of tRNAs genome-wide using the tRNA-charging assay. Among four tRNA^Ser^ isoacceptors, tRNA^Ser^_(TGA)_ showed a significant reduction upon UV-B exposure and two trended lower (AGA, GCT) (Figure 6B). These results were further supported by measurements of charged tRNA^Ser^_(TGA)_ by qRT-PCR (Figure 6C). As a control, there were no significant changes in tRNA^Asn^ charging as judged by qRT-PCR, consistent with our tRNA-charging assay measurements (Figure 6D). These correlative results suggest that lowered serine levels and the resulting lowered charging of certain tRNA^Ser^ in response to UV-B irradiation are a trigger for activation of GCN2 and translational control.

Given that activation of GCN2 during UV-B stress was accompanied by lowered levels of free amino acids, we addressed whether the supplementing the culture medium with additional amino acids suppresses activation of GCN2 by UV-B stress. Indeed, further supplementation with a full complement of amino acids sharply lowered phosphorylation of both GCN2 and eIF2α in NTERT cells exposed to UV-B irradiation (Figure 6E). In addition to protein synthesis, serine is central for intermediary metabolism and affects cellular antioxidative capacity by feeding the biosynthetic pathways for cysteine and glutathione. We found that the supplementation with the antioxidant *N*-acetyl-l-cysteine (NAC) was sufficient to lower P-GCN2 and P-eIF2α in NTERT cells following UV-B irradiation (Figure 6E). The addition of cystine, glycine, and serine, which are used in the synthesis of glutathione (48,49), collectively yielded comparable results (Figure 6F). In agreement with the idea that following UV irradiation amino acids are utilized to bolster cellular antioxidative capacity, we determined that supplementation of additional amino acids or the addition of NAC to culture media lowered UV-B induced reactive oxygen species (ROS) levels (Figure 6G). Glutathione (GSH) is an important antioxidant in cells that can help mitigate ROS. In keeping with the induced ROS following UV-B exposure, we observed that UV radiation depleted cellular glutathione (GSH) levels, which was rescued by supplementation of additional amino acids or NAC to the culture media prior to UV-B irradiation (Figure 6H). These results support the model that diminished levels of amino acids following UV-B irradiation is important for activation of GCN2, and UV-B induction of ROS is suggested to be a contributor to the processes activating GCN2.

## Discussion

This manuscript addresses two prevailing models for activating GCN2- ribosome stalling/collisions and accumulating uncharged tRNAs, in the ISR in response to diverse stresses. Our results suggest that depending on the stress condition either GCN2 process can trigger an ordered mechanism of activation requiring self-phosphorylation and the HARS-related domain. Supporting the idea that ribosome stalling/collisions can be potent inducers of GCN2, treatment with lower amounts of anisomycin activated GCN2, as viewed by increased P-GCN2 and P-eIF2α, and this induction was relieved by deletion of ZAK or by release of colliding ribosomes by pre-treatment with puromycin (Figures 1C and 2A–C). These results are consistent with a report by Wu et al. (21) that described the roles of ZAK as a direct activator of GCN2 during periods of intermediate ribosome collisions, whereas more frequent or ‘dangerous’ amounts of collisions instead induce ZAK phosphorylation of p38 (Figure 1B).

Agents that block charging of specific tRNAs, such as HF that inhibits aminoacylation of tRNA^Pro^, are suggested to instead activate GCN2 by direct GCN2 engagement with the cognate uncharged tRNAs (Figure 1D-E). In a dose-dependent fashion HF activated GCN2 (Figure 1D). The HF triggered accumulation of uncharged tRNA^Pro^ (Figures 2E, 3A–B, and Supplementary Figure S3) and tRNA^Pro^ bound to GCN2 by a mechanism requiring the function of the HARS-related domain (Figure 3C and D). The GCN2-m2 substitution, which debilitated tRNA binding (Figure 3D), blocked activation of GCN2 and its phosphorylation of eIF2α in response to HF (Figure 3C). Deletion of ZAK did not deter induction of GCN2 by HF or other agents that lower aminoacylation of tRNAs (Figure 1D, E). Furthermore, Snieckute *et al.* (50), reported that while there was appreciable ribosome stalling and activation of ZAK in livers of mice fed a leucine deprived diet, there was comparable induction of P-eIF2α in both WT and ZAK KO animals.

It is noted that high doses of HF were potent activators of GCN2 and at these levels there was appreciable ZAK-dependent P-p38 (Figure 1D), indicative of more frequent amounts of ribosome collisions. At these dangerous amounts of ribosome collision, the anisomycin treatment regimen favored ZAK phosphorylation of p38 at the expense of GCN2 (21). Arguing further against the necessity for ribosome collisions in the activation of GCN2 by HF, pretreatment with puromycin, which prematurely released nascent polypeptides and potently lowered ribosome collisions, did not affect HF reductions of tRNA^Pro^ charging or HF activation of GCN2 (Figure 2D and E). Taken together, these results support the idea that uncharged tRNAs can be direct activators of GCN2 during stress conditions that potently lower aminoacylation of tRNAs and these processes do not require ZAK or ribosome collisions.

### Multiple mechanisms can activate GCN2 depending on stress arrangements

While ZAK and ribosome stalling/collisions are suggested to be largely dispensable for activation of GCN2 in response to HF treatment, it does not preclude the idea that ribosome stalling/collisions can be an enhancer or perhaps a redundant activator of GCN2 in conjunction with HF activation by direct GCN2 binding with uncharged tRNAs. Cells depleted for ZAK showed a modest, albeit not statistically significant, lowering of P-eIF2α in response to HF (Figure 1D). Of importance treatment with velcrin, which depletes tRNA^Leu^_(TAA)_ (Figure 4A) (45), activated GCN2 and induced P-eIF2α and triggered ribosome collisions (Figure 4). Pretreatment with puromycin, lowered velcrin-induced ribosome collisions and thwarted induction of GCN2. In our previous study, we also observed that HF treatment led to ribosome stalling at proline codons (36). These results support the idea that ribosome stalling/collisions triggered by deficiencies for aminoacylated tRNAs can be a contributor to GCN2 regulation.

It is important to emphasize that the GCN2-m2 mutant blocked activation by both HF and anisomycin treatments; therefore, the HARS-related domain of GCN2 is suggested to be a common contributor to ordered mechanisms of GCN2 activation by direct engagement with tRNAs or by ribosome stalling/collisions. Together, our study suggests that multiple processes can concurrently activate GCN2 depending on stress condition. During stresses that trigger high levels of accumulating uncharged tRNAs, uncharged tRNA binding to GCN2 is likely to be a predominant activating model. By contrast, during stress conditions in which accumulating uncharged tRNAs are modest or do not appreciably occur, ribosome stalling/collisions is likely a major trigger for activation of GCN2. The ribosome stalling/collision activation of GCN2 likely involves GCN2 engagement with ribosome stalk proteins (22,23) and/or with ZAK-dependent processes (21).

The multiple mechanism hypothesis for GCN2 activation is supported by a recent study by Gupta and Hinnebusch (51) who discerned the roles of the P stalk components in the activation of GCN2 in budding yeast. By using genetic manipulations, the P stalk proteins P1/P2 were required for activation of GCN2 in response to several stress arrangements that were suggested to trigger stalling of ribosomes with empty A sites without appreciably affecting aminoacylation of tRNAs. However, genetic alterations of the P stalk proteins did not deter activation of GCN2 in response to starvation for single amino acids. This earlier report featuring the yeast model system suggested that the uncharged tRNAs and the ribosome P stalk proteins can functionally substitute for binding to the HARS-related domain, triggering an order mechanism of activation of GCN2 (51). The results of our study of mammalian GCN2 are in congruence with these ideas.

Yan and Zaher (52) also reported that stalling of yeast ribosomes by high concentrations of alkylation and oxidation agents can be potent inducers of GCN2. Lower amounts of these stress conditions can instead activate the E3 ligase HEL2 (ZNF598) that serves to ubiquitylate ribosome proteins and resolve stalled ribosomes in the quality-control pathway (RQC). In this way, activation of HEL2 would help preclude induction of GCN2 during alkylating and oxidizing conditions and only during severe stresses or the omission of HEL2 and the RQC would this eIF2α kinase be activated (52). This study highlights that GCN2 can function in conjunction with other stress sensing pathways to ensure the integrity of protein synthesis and homeostasis.

### Processes contributing to activation of GCN2 in response to UV irradiation

UV-B and UV-C irradiation are potent inducers of GCN2 and translational control (46,47). It was suggested that UV damage of mRNAs can stall translating ribosomes, leading to ribosome collisions that activate GCN2 through its engagement with ZAK (21). However, we did not observe any appreciable change in activation of GCN2 between WT cells and those knocked out for ZAK in response to increasing amounts of UV-B or UV-C exposure (Figure 5A and B and Supplementary Figure S4A–C). Furthermore, loss of ZAK did not appreciably alleviate the lowered translation initiation following UV-B treatment (Figure 5C). We are not currently clear about the reasons for these observed differences. We carried out our experiments with human keratinocytes NTERT cells and HEK293T, whereas the earlier report primarily focused UV-C exposure of human epithelial MCF10A cells (21). Along with the differences in cultured cell models, there may be variations in the UV treatment regimens, including the timing of ISR induction following UV exposure.

We further addressed the contributions of ribosome collisions for GCN2 activation in keratinocytes treated with UV-B or UV-C and determined that pre-treatment with puromycin did not have an appreciable effect on GCN2 as measured by P-GCN2 and P-eIF2α (Figure 5D and Supplementary Figure S2). We next considered the alternative uncharged tRNA model for activation of GCN2 by UV stress. We found that UV exposure lowered the levels of all free amino acids (Figure 6A). Furthermore, genome-wide measurements of charged tRNAs showed a sharp reduction in the tRNA^Ser^_(TGA)_ in response to UV-B exposure (Figure 6B–D). Supplementation of high levels of amino acids to the culture medium thwarted activation of GCN2 by UV-B, supporting the idea that diminished levels of free amino acids are contributors to activation of GCN2 (Figure 6E). In addition to protein synthesis, serine participates in intermediary metabolism and in combination with glycine contributes to biosynthetic pathways for cysteine and glutathione. Supplementation with combined cystine, glycine, and serine or NAC alone was sufficient to lower activation of GCN2 in cells following UV-B irradiation (Figure 6E and F). Addition of high amino acids or NAC also lowered the ROS levels and helped restore the glutathione (GSH) levels triggered by UV-B (Figure 6G and H). These results support the idea that ROS induced by UV-B can trigger consumption of certain amino acids. such as serine, which support cellular antioxidation by fueling the synthesis cysteine and glutathione. Loss of GCN2 and the accompanying lowered amino acids would render cells more vulnerable to UV stress (25,53). GCN2 and its maintenance of amino acids was also reported to be central for coordination of ROS in keratinocytes subjected to wounding (28). These results support the model that diminished levels of amino acids and charged tRNA is central for activation of GCN2 in response to UV stress.

## Supplementary Material

gkae006_Supplemental_File

## Data Availability

The tRNA-charging assay data generated in this study have been deposited in the NCBI’s GEO database under the accession code GSE239867. The demultiplexing python script and the analysis scripts are available on Figshare (https://doi.org/10.6084/m9.figshare.24553186.v1) and Github (https://github.com/carlsonkPhD/demultiplex). Data is presented within the manuscript, and plasmids and other reagents are available for academic purposes upon request.

## Notes

Present address: Kenneth R. Carlson, Eli Lilly and Company, Indianapolis, IN, USA.
