# Supplementary material for: Multiple mechanisms activate GCN2 eIF2 kinase in response to diverse stress conditions
Source: Nucleic Acids Res. 2024 Jan 28;52(4):1830–46. doi: 10.1093/nar/gkae006 (PMC10899773; doi:10.1093/nar/gkae006)
Supplement: gkae006_Supplemental_File [file gkae006_supplemental_file.pdf]

Supplemental Information

Multiple mechanisms activate GCN2 eIF2 kinase in response to diverse stress conditions

Jagannath Misra[1], Kenneth R. Carlson[1+], Dan F. Spandau[1,2,3], Ronald C. Wek[1*]

[1]Department of Biochemistry and Molecular Biology, Indiana University School of Medicine, Indianapolis, Indiana, USA
[2]Department of Dermatology, Indiana University School of Medicine, Indianapolis, Indiana, USA
[3]Richard L. Roudebush Veterans Administration Medical Center, Indianapolis, Indiana, USA

Running title: GCN2 activation by multiple mechanisms

Key words: Translational control, Integrated stress response, GCN2, eIF2 kinase, eIF2 phosphorylation

*To whom correspondence should be addressed: Ronald C. Wek, Department of Biochemistry and Molecular Biology, 635 Barnhill Drive, Indiana University School of Medicine, Indianapolis, Indiana 46202-5122; E-mail: rwek@iu.edu.

[+]Present address: Eli Lilly and Company, Indianapolis, Indiana, USA

Supplementary Figure 1

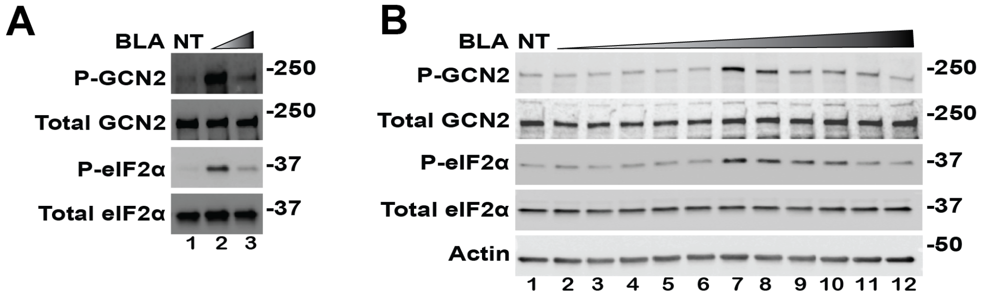

Figure S1. Activation of GCN2 by blasticidin. (A) HEK293T cells were treated with increasing amounts of blasticidin (BLA) for 3 hours. Lane 1 no treatment (NT), lane 2 (25 nM), and lane 3 (100 nM). Protein lysates were prepared and the amounts of the indicated phosphorylated and total proteins were measured by immunoblot analyses. MW markers are indicated in kDal. **(B)** MEF cells were treated with increasing amounts of blasticidin (BLA) for 3 hours. Lane 1 no treatment (NT), lane 2 (5 nM), lane 3 (50 nM), lane 4 (100 nM), lane 5 (250 nM), lane 6 (500 nM), lane 7 (750 nM), lane 8 (1 µM), lane 9 (50 µM), lane 10 (100 µM), lane 11 (250 µM), and lane 12 (500 µM). Protein lysates were prepared and the amounts of the indicated phosphorylated and total proteins were measured by immunoblot analyses.

Supplementary Figure 2

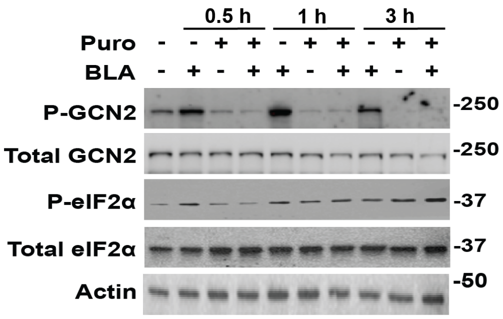

Figure S2. Puromycin thwarts GCN2 activation by blasticidin. (A) MEF cells were treated with 750 nM blasticidin (BLA) and or 1 µM puromycin (Puro) for the indicated times, as indicated by the - and + symbols. Protein lysates were prepared and the levels of the indicated phosphorylated and total proteins were measured by immunoblot analyses. MW markers are indicated in kDal.

Supplementary Figure 3

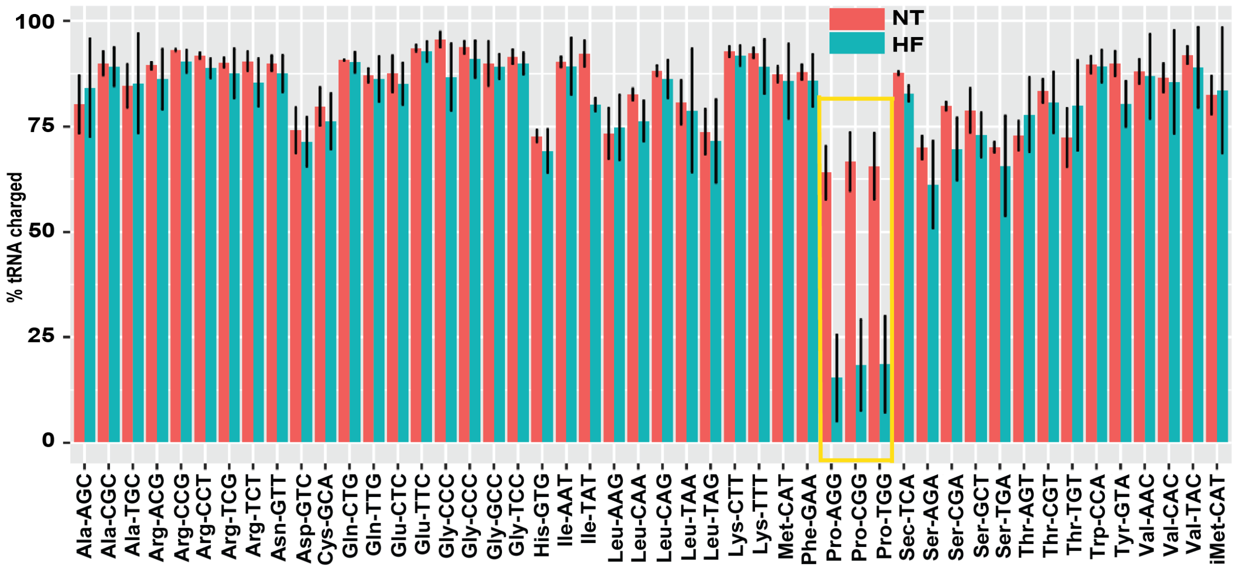

Figure S3. HF treatment of MEF cells specifically reduces the charging of tRNA^{Pro}. Genome-wide tRNA-charging measurements of MEF cells were left untreated (NT) or treated with 100 nM HF or not treated (NT) for 3 hours. The percentage of charging for the indicated isoacceptor tRNAs is shown as a bar graph. The error bars represent the SD of the mean for n=3 biological replicates. Statistical significance was determined by two-sided Welch's t-test followed by correction for multiple hypothesis testing using Benjamini-Hochberg FDR method. The yellow box indicates the tRNA^{Pro} isoacceptors that have reduced charging in MEF cells treated with HF.

Supplementary Figure 4

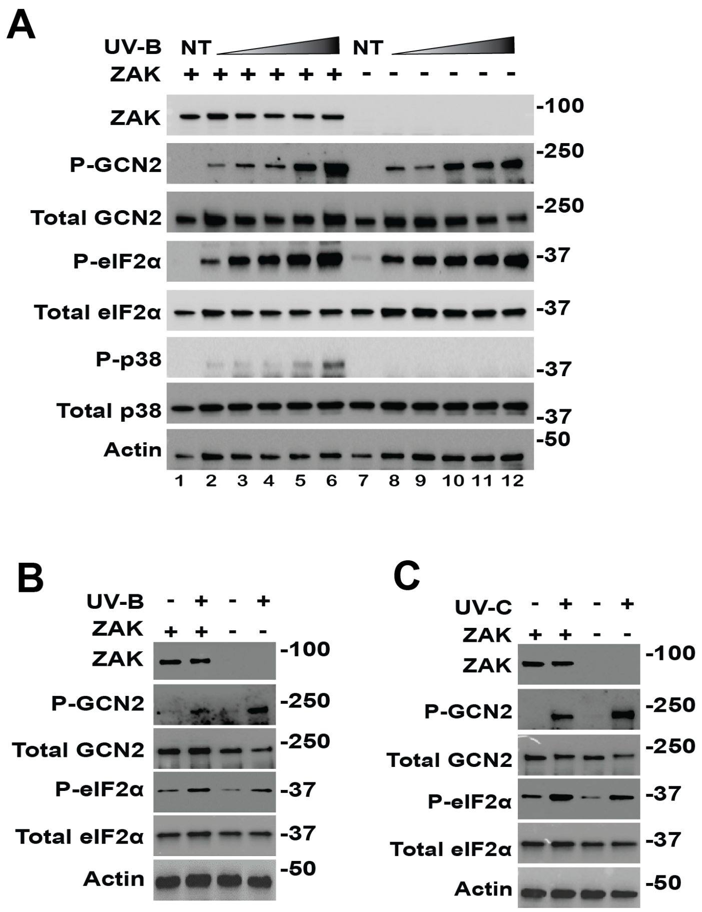

Figure S4. UV activation of GCN2 is blocked by puromycin. (A) WT (+) and ZAK KO (-) HEK293T cells were treated with increasing doses of UV-B (200-1000 J/M^2) or left untreated (NT), followed by recovery for 3 hours. Lanes1 and 7 (0 J/M^2), 2 and 8 (200 J/M^2), 3 and 9 (400 J/M^2), 4 and 10 (600 J/M^2). 5 and 11 (800 J/M^2), and 6 and 12 (1,000 J/M^2). Protein lysates were prepared and the indicated phosphorylated and total proteins were measured by immunoblot. **(B-C)** WT (+) and ZAK-deleted (-) NTERT cells were treated 400 J/m^2 UV-B **(B)** or 1200 J/m^2 UV-C **(C)** or left untreated (NT), followed by recovery for 30 minutes. Protein lysates were prepared and immunoblot analyses were used to measure the levels of the indicated phosphorylated and total proteins.
